# Editorial: Epidemiology and control of emerging/re-emerging poultry and waterfowl diseases

**DOI:** 10.3389/fmicb.2026.1841273

**Published:** 2026-04-15

**Authors:** Weiwei Wang, Jiafeng Wu, Matteo Legnardi, Tianchao Wei, Chunhe Wan, Ping Wei, Yu Huang

**Affiliations:** 1 Institute of Animal Husbandry and Veterinary Medicine, Fujian Academy of Agricultural Sciences (FAAS)/Fujian Industry Technology Innovation Research Academy of Livestock and Poultry Diseases Prevention and Control, Fujian Academy of Agricultural Sciences (FAAS)/Fujian Key Laboratory for Control and Prevention of Avian Diseases, Fuzhou, China; 2 Institute for Poultry Science and Health, College of Animal Science and Technology, Guangxi University, Nanning, China; 3 Department of Animal Medicine, Production and Health (MAPS), University of Padua, Legnaro, PD, Italy

**Keywords:** poultry and waterfowl diseases, epidemiology and control strategies, *Riemerella anatipestifer* (RA), duck hepatitis A virus type 3 (DHAV-3), avian leukosis virus subgroup J (ALV-J), *Aspergillus*, *Mycoplasma gallisepticum* (MG), *Salmonella*

In recent years, rapid expansion of the global poultry and waterfowl industries has been accompanied by increasing reliance on high-density and intensive production systems. While these systems improve productivity, they also create favorable conditions for pathogen transmission, persistence, and evolution. Viral and bacterial infections remain leading causes of disease outbreaks and economic loss in poultry, and their epidemiology has become increasingly complex owing to genetic variation, antimicrobial resistance (AMR), host adaptation, and frequent co-infections. In this situation, the Research Topic brings together nine studies addressing molecular epidemiology, genomic characteristics, host responses, and control strategies of important emerging and re-emerging poultry and waterfowl diseases.

A central theme of the Research Topic is the changing epidemiology and host adaptability of bacterial pathogens. Several contributions focus on *Riemerella anatipestifer* (RA), a pathogen traditionally used to be associated with ducks and geese but now increasingly be recognized in chickens. Zhang et al. investigated the biological and genomic characteristics of chicken-derived RA in China. Serotypes 1 and 10 were predominant, with serotype 10 showing higher virulence in chicken embryos. Both serotypes caused clear clinical symptoms in SPF chickens and exhibited tropism for joints and the central nervous system. Lv et al. demonstrated that strains of serotypes 1, 5, and 10 could induce similar clinical manifestations in hens of different ages. Comparative genomic analysis between chicken- and duck-derived strains identified 18 mutated virulence genes, including those related to type IV secretion systems, hemolysin, lipooligosaccharide/lipopolysaccharide synthesis, capsule biosynthesis, and iron acquisition systems. Host determinants of disease susceptibility are another important focus of this Research Topic. Zou et al. investigated different resistance to RA infection in commercial White Kaiya ducks (WK) and native Ji'an Red-feathered ducks (JR) using transcriptomic and metabolomic analyses. Resistant JR ducks maintained immune–iron homeostasis, restricting iron availability while activating immune responses, whereas susceptible WK ducks showed disrupted iron metabolism and excessive inflammation. These findings highlight the key role of immune–iron homeostasis may be an important determinant of resilience to RA infection.

Viral evolution and pathogenesis are another central pillar of this Research Topic. Fu et al. compared liver transcriptomic responses in ducks infected with virulent or attenuated duck hepatitis A virus type 3 (DHAV-3). The virulent strain induced markedly stronger activation of pattern-recognition receptor pathways, interferon-related responses, and inflammatory signaling cascades than the attenuated strain. These results offer important mechanistic insight into the balance between protective innate immunity and immunopathology during DHAV-3 infection, and that may help explain the biological basis of attenuation. Complementing this work, Dan et al. described the isolation of a potentially novel DHAV-3 strain, HNAY2024, from a vaccinated duck flock in China. This strain caused disease in older ducklings than typically expected and contained eight amino acid substitutions (V413M, E683Q, V855I, F892S, S1149I, T1151S, E1519G, and K1956E), raising concern about ongoing antigenic drift and possible vaccine escape. Together, these studies underscore the continued evolution of DHAV-3 and the need for sustained genomic surveillance and timely adjustment of vaccination strategies.

The Research Topic also highlights the continued circulation and evolution of avian leukosis virus subgroup J (ALV-J) in native chickens. Liu et al. reported the molecular characterization of recent ALV-J isolates from Chinese native chicken breeds revealed the persistence of multiple clades and identified mutations in the envelope gene and 3′ UTR that may affect replication, pathogenicity, and persistence. These findings indicate that local chicken populations may remain important reservoirs for the continued evolution of avian retroviruses, even where control has been improved in commercial breeding systems.

Beyond conventional production-associated pathogens, this Research Topic also extends to wildlife and fungal disease ecology. A study reported by Rivelli Zea et al. of aspergillosis in endangered Okinawa rails identified *Aspergillus terreus* and *Aspergillus pseudonomiae* as causative agents in captive birds. Particularly noteworthy was reduced voriconazole susceptibility in one *A. terreus* isolate and the recovery of two phenotypically distinct *A. pseudonomiae* isolates from the same host, one of which had lost both sclerotia formation and aflatoxin production. These observations show that fungal pathogens may diversify within hosts and highlight challenges for wildlife conservation, captive management, and anti-fungal stewardship.

Novel control options are another unifying topic across the Research Topic. Zidi et al. evaluated Spirulina platensis as a natural antimicrobial candidate against macrolide-resistant *Mycoplasma gallisepticum* (MG). *In vitro*, Spirulina showed inhibitory activity against many field isolates, including multidrug-resistant strains, while displaying low cytotoxicity. Although *in vivo* validation and mechanistic studies are still needed, this work points to the potential value of alternative or complementary antimicrobial strategies as the conventional drug efficacy declines under AMR pressure.

The public health relevance of poultry-associated diseases is clearly represented by the study using chick paper sampling during multistate Salmonellosis outbreaks linked to backyard poultry in the United States. By testing contaminated shipping materials at points of sale, investigators successfully detected outbreak-associated *Salmonella* strains and improved traceback to source hatcheries. Zlotnick et al. provides a practical example of a One Health approach integrating animal health, environmental sampling, epidemiological investigation, and public health response. It also illustrates how low-cost field sampling tools can strengthen outbreak detection and intervention across interconnected production and household settings.

Finally, the studies included in the Research Topic collectively highlight the increasing complexity of emerging and re-emerging poultry and waterfowl diseases, driven by pathogen evolution, antimicrobial resistance, and host-pathogen interactions. The integration of molecular epidemiology, genomics, and novel control strategies provides valuable insights for improving disease surveillance, diagnosis, and intervention. Continued interdisciplinary efforts and the application of innovative technologies will be essential to effectively mitigate disease risks and support the sustainable development of the poultry industry.

